# Unveiling cost reduction potential of carbon capture and storage clusters in China under multiple uncertainties

**DOI:** 10.1016/j.isci.2025.113315

**Published:** 2025-08-06

**Authors:** Yaxian Wang, Jizhe Li, Xian Zhang, Kai Li, Jing-Li Fan, Xiaojuan Xiang, Yunbing Hou

**Affiliations:** 1 Centre for Sustainable Development and Energy Policy Research, School of Energy and Mining Engineering, China University of Mining and Technology (Beijing), Beijing 100083, China; 2 The Administrative Center for China’s Agenda 21, Beijing 100038, China; 3 Department of Earth System Science, Ministry of Education Key Laboratory for Earth System Modeling, Institute for Global Change Studies, Tsinghua University, Beijing 100084, China

**Keywords:** Environmental science, Energy sustainability, Energy storage

## Abstract

Carbon capture and storage (CCS) plays a pivotal role in China’s low-carbon power transition but faces high costs and multiple uncertainties related to technology, policy, and economics. CCS clusters, along with the associated hubs, offer an opportunity to tackle this issue. Therefore, a CCS cluster source-sink matching model was developed based on fuzzy possibility programming and interval linear programming methods under multiple uncertainties. The cluster layout, shared pipeline network, and cost reduction potential were explored. Results show that the North China, Northwest China, East China, and South China regions will be prioritized for the development of CCS cluster projects. Compared to “point-to-point” projects, CCS clusters have great potential to reduce pipeline lengths by [81.5%, 81.8%], and lower the plant-level levelized cost of CCS by [35.7%, 86.9%]. This study provides a system-wide insight for CCS cluster development and cost reduction assessment under multiple uncertainties.

## Highlights

• Developed a CCS cluster model under multiple uncertainties
• North, Northwest, East, and South China are priority regions for CCS cluster development
• CCS clusters cut pipeline lengths by [81.5%, 81.8%] compared with point-to-point projects
• CCS clusters cut plant-level LCOC by [35.7%, 86.9%] compared with point-to-point projects

## Graphical abstract

**Figure:**
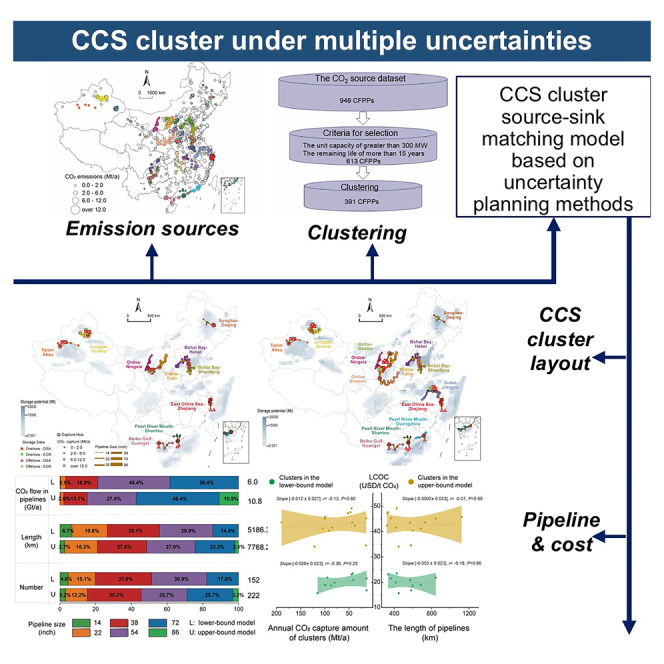

## Introduction

Urgent climate action is critically needed due to the continued increase in carbon emissions.^1^ A global consensus has been reached on the objective of holding the increase in the global average temperature to well below 2°C above pre-industrial levels and to pursue efforts to limit warming to 1.5°C. China has announced its ambitious goal of achieving carbon neutrality by 2060. The power system accounts for nearly 50% of China’s energy-related CO_2_ emissions,^2^^,^^3^ making its low-carbon transformation critical to meeting these ambitions.^4^ Carbon capture and storage (CCS) is essential for achieving emission reductions and serves as a vital tool for decarbonizing the power system. With increasing climate ambitions, CCS technology has developed rapidly in recent years. However, growth and investment in the CCS industry have fallen short of expectations, primarily due to high investment costs, multiple uncertainties (such as policy, economic, technological, and storage prospects).^5^

**Figure 1:**
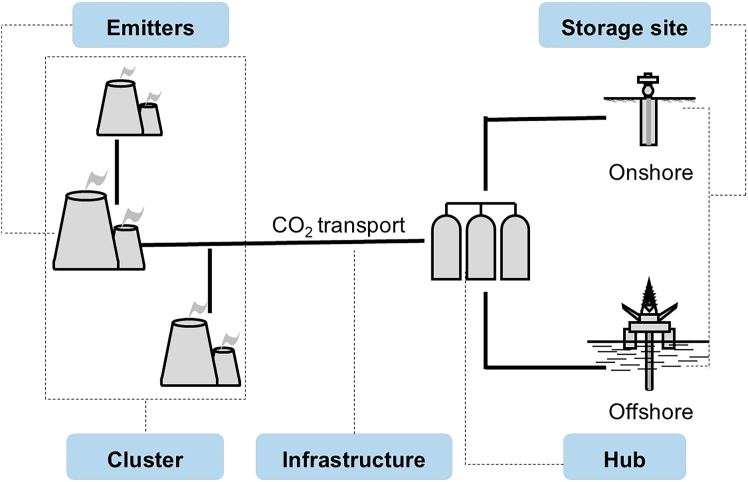
Diagram of CCS clusters and hubs *Note:* CCS clusters are geographically concentrated emitters; Infrastructure refers to the shared CO₂ transportation network consisting of pipelines, trucks, or ships. CCS hubs are the central collection or distribution points for CO_2_.^8^

CCS clusters, along with the associated hubs, offer an opportunity to tackle this issue.^6^^,^^7^ The cluster consists of several projects that can capture CO_2_ from multiple emission sources, utilize shared infrastructure (such as pipelines), and storage networks for CO_2_ (Figure 1).^8^ A hub can facilitate the collection of CO_2_ from a capture cluster or the distribution of CO_2_ to a storage cluster.^9^ The global investment in CCS technology has surged, with a marked shift toward the development of clusters and hubs. Globally, over 40 CCS clusters or hubs are operational or under construction,^10^ such as the Northern Lights/Longship in Norway,^11^ the Aramis project in the Netherlands,^12^ and the Junggar Basin project in northwest China.^13^

Emission reduction demands, technological developments, and mitigation-related policies have increasingly amplified uncertainties, presenting significant challenges to the deployment of CCS (clusters).^14^ In developing CCS (clusters), investors need to consider many factors such as economic, technical, and political issues. For example, deep uncertainty about sustainable injection rates at storage sites constrains both the scale and pace of CCS deployment.^5^ On the policy side, over 150 countries have pledged carbon neutrality, most lack concrete policy roadmaps,^15^ and there are also large uncertainties about the demand for emission reductions and the effects of climate policy interventions.^16^ Meanwhile, CCS technologies are still under development with high variability in cost parameters.^17^^,^^18^ Therefore, these multiple uncertainties need to be quantified in the model. Substantial efforts have been made to evaluate the emission reduction potential,^19^ storage prospects,^20^^,^^21^^,^^22^ cluster layouts,^23^^,^^24^^,^^25^ transmission pipeline networks,^26^ and costs^27^^,^^28^ with spatial optimization models, such as the source-sink matching model—a computational framework designed to optimize the connection between CO_2_ emission sources and storage sites. Most previous studies tend to model the system parameters as deterministic inputs, such as capital costs, injection rates, and emission reduction demands, thereby simplifying the complexities of real-world cases. Recent studies have employed methods, such as real options^29^^,^^30^ and Monte Carlo simulations,^31^ to deal with stochastic uncertainties. However, most focus on a single “point-to-point” project,^9^^,^^32^ and some parameters are more appropriately characterized by fuzzy or interval uncertainties.^33^ The limited evaluation of uncertainties in CCS clusters may result in deviations between model predictions and real-world conditions.

CCS clusters are widely regarded as a cost-effective pathway,^6^^,^^7^^,^^34^ but their economic performance under multiple uncertainties has not been comprehensively assessed, potentially hindering the reliability of decision-making. Moreover, under such uncertain conditions, the layout of the CCS cluster and its pipeline networks remains unclear. For example, most existing studies focus on source-sink (or hub-storage site) spatial matching, with limited attention to the design and cost optimization of pipeline networks between emission sources within clusters. The geographical center of a cluster is often assumed to serve as the capture hub,^35^^,^^36^ which is not in line with the idea of minimizing the full-chain costs. This study explores the CCS cluster layout, shared pipeline network, and cost-effectiveness by developing a CCS cluster source-sink matching model that incorporates real-world uncertainties. The model includes 946 coal-fired power plants (CFPPs), 17 main basins at storage sites (40 km × 40 km) involving onshore/offshore enhanced oil recovery (EOR) and deep saline aquifers (DSA). We quantify the multiple uncertainties, e.g., fuzzy and interval, and construct a more realistic dataset to elucidate the impacts of these uncertainties on the spatiotemporal evolution of CCS clusters. By integrating fuzzy possibility programming (FPP) and interval linear programming (ILP) methods, the model addresses uncertainties in parameter inputs. Meanwhile, the hub selection mechanism is optimized and incorporates hierarchical pipeline network planning within the cluster. Finally, the economic feasibility of CCS clusters is assessed, offering a strategic solution to facilitate the low-carbon transition of China’s power system under real-world conditions.

## Results

### Carbon capture and storage cluster layout under multiple uncertainties

**Figure 2:**
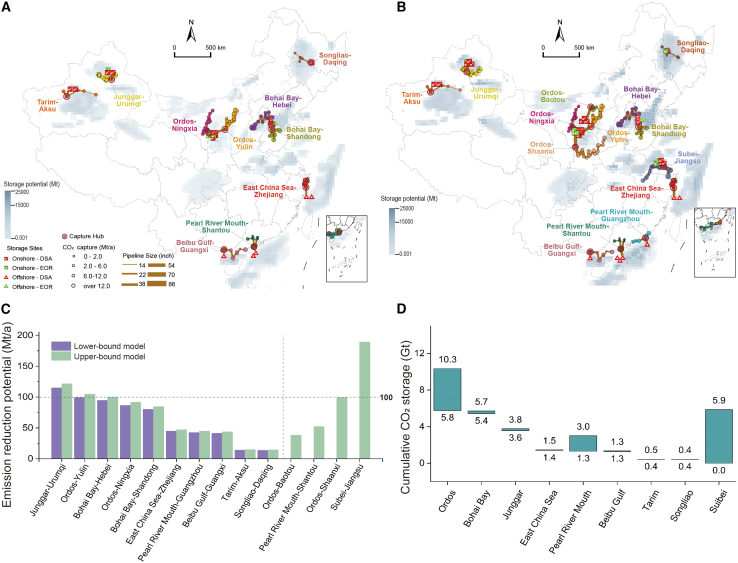
CCS cluster layout and emission reduction potential under multiple uncertainties (A and B) CCS cluster layout under the lower-bound (A) and upper-bound (B) models, with a 500 km scale bar included. (C) Emission reduction potential of CCS clusters under multiple uncertainties. (D) Cumulative storage under multiple uncertainties. *Note:* The cluster source-sink matching model integrates fuzzy possibility programming (FPP) and interval linear programming (ILP) through a two-stage solution approach. Specifically, the model is decomposed into a lower-bound model and an upper-bound model based on the treatment of uncertainties. The lower-bound model incorporates the loosest set of constraints, reflecting optimistic assumptions and yielding the minimum objective value. In contrast, the upper-bound model applies the tightest constraints, reflecting conservative assumptions and producing the maximum objective value. Data are represented as intervals based on upper- and lower-bound results under multiple uncertainties.

The cumulative emission reduction demands of China's power system is estimated to be 19.6–32.4 Gt CO_2_ from 2030 to 2060.^37^^,^^38^ 10 clusters are prioritized for development, with additional 4 clusters considered as candidates if emission reduction demands continue to increase (Figures 2A and 2B). The CCS clusters are generally located in North China, Northwest China, East China, and South China regions, with the emission reduction potential of [280.5, 334.2] Mt/a, [129.0, 235.4] Mt/a, [124.9, 321.1] Mt/a, and [83.5, 140.3] Mt/a considering uncertainties in policies, technologies, and economics (Figure 2C). When emission reduction demands increase to 32.4 Gt, 79 CFPPs, located in Ordos-Baotou, Pearl River Mouth-Shantou, Ordos-Shaanxi, and Subei-Jiangsu regions, can be further retrofitted to satisfy these demands.

Among these clusters, the emission reduction potential of Junggar-Urumqi, Ordos-Yulin, and Subei-Jiangsu clusters is particularly remarkable, each exceeding 100 Mt/a, with Subei-Jiangsu cluster standing out at 189 Mt/a (Figure 2C). This cluster features a high density of CFPPs along the Yangtze River, most of which have substantial carbon emissions, is well-positioned to effectively integrate these sources for large-scale decarbonization.

The Ordos Basin, the Bohai Bay Basin, and the Junggar Basin are the main storage sites, offering exceptional geological storage suitability (Figure 2D). During the time period from 2030 to 2060, the cumulative storage potentials are estimated at [5.8, 10.3] Gt, [5.4, 5.7] Gt, and [3.6, 3.8] Gt CO_2_, respectively. When emission reduction demands increase to 32.4 Gt, the Subei Basin, the Ordos Basin, and the Pearl River Mouth Basin will play a significant role in large-scale CO_2_ storage, offering a potential of 5.9 Gt, 4.5 Gt, and 1.7 Gt, respectively. Regarding the type of storage site (Table S1), the CO_2_ storage method through EOR generates benefits by improving oil recovery rates while achieving CO_2_ sequestration. But it is limited by storage capacity. DSA enables effective CO_2_ sequestration with significant storage potential. In the upper-bound model, 24 storage sites are included, of which 23 are DSA sites with a storage capacity of 31.8 Gt, representing 98.1% of the total storage capacity. In comparison, the lower-bound model involves 45 storage sites, requiring only 11 DSA sites to achieve 99.4% of the total capacity (19.5 Gt).

### Shared pipeline network of carbon capture and storage clusters under multiple uncertainties

**Figure 3:**
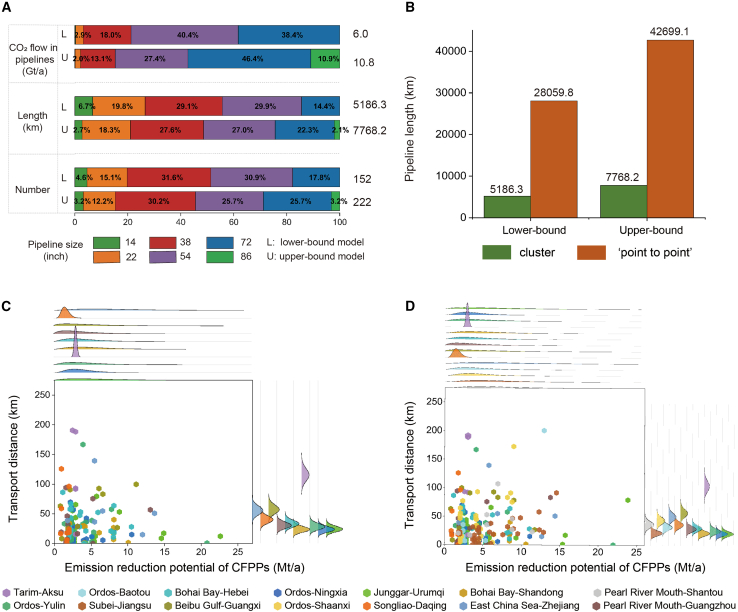
Characteristics of shared pipelines in CCS clusters under multiple uncertainties (A) Proportions of different pipelines. (B) Comparison of pipeline length between clusters and the “point-to-point” projects. (C and D) CO_2_ capture and transport distances of lower-bound (C) and upper-bound (D) models. Note: Data are represented as intervals based on upper and lower bound results under multiple uncertainties.

More than 5000 km (152 pipelines) of shared pipelines need to be constructed for transporting CO_2_ from emission sources to storage sites, meeting the emission reduction demands for 19.6 Gt. The length and number of pipelines will increase by 49.8% and 46.1%, respectively, reaching 7768.2 km and 222 pipelines when emission reduction demands increase to 32.4 Gt (Figures 3A and S1). Trunk pipelines with diameters exceeding 30 inches are pivotal in CCS cluster projects, the length accounts for [73.5%, 79.0%] of the total, representing an increase of [21.5%, 27.0%] compared to the “point-to-point” source-sink matching results in CCS retrofits for CFPPs.^26^ This shift reflects the clustering effect, whereby CO_2_ flows in trunk pipelines instead of dispersing them across smaller ones. In cluster networks, each pipeline carries the total CO_2_ captured from all upstream sources, transporting CO_2_ at [6.0, 10.8] Gt/a. Within this framework, trunk pipelines carrying [96.8%, 97.7%] of the total flow. CCS clusters significantly shorten transportation distances (Figure 3B). Based on current source-sink matching, connecting each source to its sink individually would require [28059.8, 42699.1] km of pipelines. The cluster approach reduces pipeline lengths by [81.5%, 81.8%], demonstrating its efficiency in optimizing the network.

CO_2_ emission reduction potential and transportation distance present considerable regional heterogeneities, considering the multiple uncertainties related to policies, technologies, and economics (Figures 3C and 3D). Regions with dense distributions of larger emitters and proximity to storage sites are more suitable for cluster development, such as the Junggar-Urumqi cluster with nearly half of the CFPPs having a transport distance of less than 50 km from each other, and average emission reduction potential of CFPPs [6.1, 6.4] Mt/a exceeding the mean value of [4.2, 4.5] Mt/a (Methods S1. Clustering process for coal-fired power plants and geographical distribution, related to STAR★Methods).

Due to the high costs, CCS retrofits of small emitters were previously unviable, but the clustering approach now makes their participation feasible and effective. For the Songliao-Daqing cluster, which has relatively small CFPPs, the average CO_2_ emission reduction potential is less than 1.6 Mt/a. With this approach, more than half of the CFPPs have transport distances within 50 km and up to 150 km. This brings significant economic benefits and improves the overall feasibility of CCS implementation for smaller emitters.

### Cost-effectiveness of carbon capture and storage clusters under multiple uncertainties

**Figure 4:**
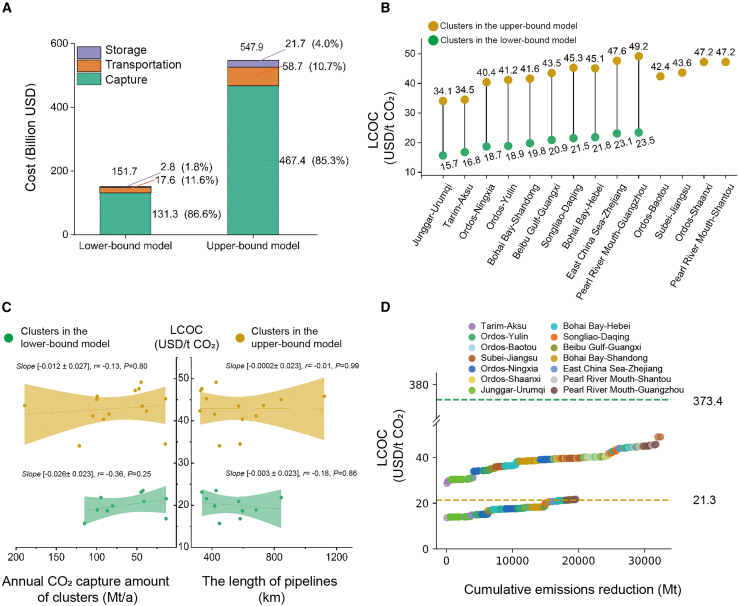
Total cost, levelized cost of CCS (LCOC), and drivers (A) Total cost of CCS clusters deployment under multiple uncertainties. (B) LCOC of CCS clusters. (C) Factors correlated with LCOC in CCS clusters. (D) LCOC of CFPPs. *Note:* Data are represented as intervals based on upper and lower bound results under multiple uncertainties; Figure C illustrates the relationships between LCOC and cluster annual capture amount or pipeline length. The continuous lines represent the fitted regression results, with the *slope,* pearson’s *r* and *p*-value of each regression displayed to indicate both the direction and the statistical significance of the correlations.

The total costs of CCS cluster projects in China’s power system exhibit a significant difference of [151.7, 547.9] billion USD, considering the multiple uncertainties related to policies, technologies, and economics (Figure 4A). Although capture costs dominate (accounting for [85.3%, 86.6%]), total CCS project investment can be significantly reduced by ensuring the optimal design and operation of transport facilities and storage sites. Transportation and storage costs can be reduced by [73.7%, 93.3%] compared to those in existing studies.^36^^,^^39^

The levelized cost of CCS (LCOC)^40^^,^^41^ of the clusters is affected to varying degrees by multiple uncertainties related to policies, technologies, and economics, yet it still demonstrates superior cost-effectiveness (Figure 4B). In the lower-bound model, the LCOC is [15.7, 23.5] USD/t CO_2_, which is growing by [105.3%, 117.9%] as the emission reduction demands increase to 32.4 Gt. Except for the Junggar-Urumqi and Tarim-Aksu clusters, all clusters exhibit a change in LCOC of more than 20 USD/t. Nevertheless, they remain economically feasible, with the potential to reduce LCOC to below 20 USD/t CO_2_, making them strong candidates for prioritized development. Increasing the capture capacity and pipeline length have limited effects on LCOC (Figure 4C). Pearson correlation analysis shows weak negative correlations between LCOC and cluster annual CO_2_ capture amount (Pearson’s r = [-0.13, -0.36], *P* > 0.05), while the correlations with pipeline length are negligible, while the correlations with pipeline length are negligible (Pearson’s r = [-0.01, -0.18], *P* > 0.05).

Clustering results indicate plant-level LCOC ranging from [13.8, 49.0] USD/t, representing a reduction of [35.7%, 86.9%] compared to previous “point-to-point” projects,^26^^,^^42^^,^^43^^,^^44^ despite being influenced by multiple uncertainties related to policies, technologies, and economics (Figure 4D). Variation in LCOC within clusters is substantial: Beibu Gulf-Guangxi, Junggar-Urumqi, and Ordos-Yulin are strongly affected by uncertainties, with LCOC being approximately 28.3, 30.1, and 27.7 USD/t (Table S2). In the lower-bound model, the plant-level LCOC is [13.8, 22.0] USD/t CO_2_, which grows by [107.2%, 122.7%] when the emission reduction demand increases to 32.4 Gt. Among these, the Aral Singhukogan coal-fired power plant exhibits the lowest LCOC and is relatively unaffected by multiple uncertainties, making it a prime candidate for priority retrofit.

## Discussion

Cluster deployment is a crucial approach to reducing CCS costs, yet it is subject to multiple uncertainties related to policies, technologies, and economics. This study presents a structured method that integrates FPP and ILP into a CCS cluster source-sink matching model, generating a bounded solution space that captures both optimistic and conservative scenarios. The analysis indicates that uncertainty can cause total investment costs to range from [151.7, 547.9] billion USD, with plant-level LCOC fluctuating by up to 117.9%. Figure S2 and Table S2 provide detailed information on plant-level cost variations under different uncertainty scenarios. Although transport and storage costs account for a smaller share ([13.4%, 14.7%]), clustering achieves cost savings of [73.7%, 93.3%] compared with traditional “point-to-point” projects. To meet the demand for emission reductions, [10, 14] CCS clusters need to be deployed. To investigate cluster characteristics and future deployment strategies, they were divided into three categories based on significant differences in emission reduction potential, shared pipeline networks, and cost-effectiveness (Figure S2).

Category 1 clusters are those with significant economic advantages and large-scale emission reduction potentials. This group includes the Ordos-Ningxia cluster, the Junggar-Urumqi cluster, the Ordos-Yulin cluster, the Bohai Bay-Shandong cluster, and the Bohai Bay-Hebei cluster. In reality, the CCS clusters around the Junggar Basin, the Ordos Basin, and the Beibu Gulf Basin are under active preparation.^45^ The cluster development potential of the above regions is confirmed by this study, and the real-life deployments further reinforce the feasibility and application value of the findings. As the largest land-based cluster, the Junggar-Urumqi cluster should fully harness economies of scale. Planning and design of a shared CO_2_ transportation and storage network should be prioritized. This would help address the current lack of large-scale CO_2_ pipeline infrastructure and the unsupportive retrofitting and utilization of existing facilities.^45^ Policy support packages are critical to the successful deployment of projects, and the effectiveness of policy mixes often exhibits regional heterogeneity.^16^ Therefore, regional governments can develop more targeted support measures within existing policy frameworks such as the “1 + N” strategy,^46^ taking into account local power system characteristics and regional economic conditions.

Category 2 clusters are characterized by relatively low economic advantages and smaller emission reduction potential (<50 Mt/a), but they remain economically viable. This group includes the Tarim-Aksu cluster, Songliao-Daqing cluster, Beibu Gulf-Guangxi cluster, and Pearl River Mouth-Guangzhou cluster. Clusters in this category exhibit slightly higher LCOC, but are still economically feasible. The cluster mode can reduce the LCOC of the Pearl River Mouth-Guangzhou cluster to below 40 USD/t CO_2_, despite the economic challenges posed by offshore projects, such as limited space, high capital investment, and complex maintenance requirements. Given the characteristics of this category, clusters can leverage the cost-effectiveness of sharing mechanisms. Promoting cooperation among clusters can reduce the duplication of investment and improve the utilization of infrastructure. For example, the geographical proximity of the Beibu Gulf-Guangxi industrial cluster and the Pearl River Mouth-Guangdong industrial cluster presents an opportunity for coordinated development. Moreover, CCS technology still faces technological bottlenecks in certain links, and its commercialization integration lags behind.^47^ This category of clusters should focus on cutting-edge and disruptive CCS technology applications to build an economically efficient and environmentally sustainable full-chain CCS technology system.

Clusters with increased retrofitting due to multiple uncertainties related to policies, technologies, and economics are classified in the third category. Deployment opportunities for clusters in this category also need to be assessed, and proactive strategies for coping with large-scale CO_2_ mitigation should be developed in advance. When the emission reduction demand increases to 32.4 Gt, the Ordos-Baotou cluster and the Ordos-Shaanxi cluster can swiftly commence the project preparation and plan, drawing on the mature experience of surrounding clusters (e.g., the Ordos-Yulin cluster) in terms of technology application, infrastructure planning, and business models.

### Limitations of the study

This study has limitations that call for further refinement in future research. While current efforts focus primarily on the electricity system, achieving future climate goals will require coordinated action across all sectors of society. Future analyses should also include emitters from other hard-to-abate sectors, such as iron and steel, cement, and chemicals. Second, carbon dioxide removal technologies^48^(e.g., direct air capture and biomass with carbon capture and storage) and geological sequestration options^49^(e.g., depleted oil and gas reservoirs, enhanced coal bed methane recovery, and others) were not included in this study. The exclusion was due to a lack of maturity, weak popularity, or data limitations. Moreover, the design and deployment of CO_2_ transport networks are influenced by regional factors such as geographic conditions and population density. Future models should consider these spatial constraints and examine phased deployment strategies to better reflect real-world implementation pathways.

## Resource availability

### Lead contact

Further information and requests for resources and reagents should be directed to and will be fulfilled by the lead contact, J.L.F. (fjlldq@163.com).

### Materials availability

This study did not generate new unique materials.

### Data and code availability

• Data: The data reported in this study are available from the lead contact on reasonable request.
• Code: This article does not report original code.
• Any additional information required to reanalyze the data reported in this article is available from the lead contact upon reasonable request.

## Acknowledgments

This study is supported by the National Natural Science Foundation of China (No. 42375173 and 72174196) and the National Postdoctoral Researchers Funding Program (GZC20231327).

## Author contributions

Y.X.W., data curation, methodology, software, formal analysis, and original draft; J.Z.L., investigation, data curation, methodology, original draft, and funding acquisition; X.Z., conceptualization and software; K.L., validation, review, and editing; J.L.F., supervision, review, funding acquisition, and project administration; X.J.X., methodology, and validation; Y.B.H., validation, review, supervision, and funding acquisition.

## Declaration of interests

The authors declare no competing interests.

## STAR★Methods

### Key resources table

**Table:** 

REAGENT or RESOURCE	SOURCE	IDENTIFIER
**Software and algorithms**		

Gurobi 12.0.0	GUROBI OPTIMIZATION, LLC	https://www.gurobi.com/
Python 3.12.4	Python Software Foundation	https://www.python.org/
Origin 2024b	Originlab	https://www.originlab.com/
ArcGIS Desktop 10.8.2	Environmental Systems Research Institute, Inc.	https://www.esri.com/en-us/home

### Experimental model and study participant details

This study did not involve any experimental models or human participants.

### Method details

The CCS cluster source-sink matching model fully accounts for multiple uncertainties related to policies, technologies, and economics (Figure S3). First, CCS clusters are identified by using the OPTICS algorithm^50^ (Figure S4; also see Methods S2. Optimal layout for CO_2_ transportation pipeline networks, related to STAR Methods). A modified minimum spanning tree algorithm (MST) is applied to calculate the transportation cost within the cluster when different emission sources act as hubs, full accounting for the impact of hub selection on the total distance between power plants. The developed model is used to assess the impact of multiple uncertainties on the least-cost layout, shared pipeline network, and economics of the full-chain of CCS clusters. This model offers vital scientific evidence for achieving cost-effective CCS deployment in pursuit of low-carbon goals.

#### Modeling assumptions

(1) Current carbon capture methods are divided into three main categories: pre-combustion capture, oxy-fuel combustion, and post-combustion capture. In this study, post-combustion capture using an amine-based absorber is assumed.
(2) The large-scale deployment of CCS infrastructure in China is assumed completed before 2030. CFPPs with a unit capacity greater than 300 MW and a remaining lifetime of more than 15 years are screened as candidate emission sources. Once the CCS retrofit is completed, its operation status and energy consumption pattern remain unchanged.
(3) The storage grid size is 40 km × 40 km. Although the Chinese government has not enacted laws or regulations on CO_2_ storage, this study assumes a maximum of 50 injection wells per grid based on engineering practices to ensure the safety of underground CO_2_ diffusion.^51^
(4) All CO_2_ captured from emission sources is asuumed to be transported to the sinks for storage without loss. Therefore, the amount of CO_2_ captured at each source is equivalent to the amount entering the pipeline from that source.
(5) Priority is given to matching highly suitable basins, taking into account the assessment of the suitability of storage sites as reported in ref.^52^
(6) The construction diameters of pipelines are 14, 22, 38, 54, 70, and 86 inches, based on the maximum diameter of the Russia- China gas pipeline east line project with appropriate adjustments.^26^ Pipelines with a diameter exceeding 30 inches are classified as trunk pipelines.

#### Objective function and constraints

The CCS cluster source-sink matching model integrates FPP and ILP to address multiple uncertainties arising from policies, technologies, and economics. ILP can handle uncertain information in the form of intervals. Both the objective function and the decision variables are expressed as interval numbers, containing upper and lower bounds. Such as, $a^{\pm }$ is defined as an interval with known upper and lower bounds but unknown distribution information. Formally, it can be represented as $a^{\pm }=\left[a^{-},a^{+}\right]=\left\{t\in a|a^{-}\leq t\leq a^{+}\right\}$. Parameter uncertainty is passed on to the optimization process, resulting in an interval solution. The objective function of this study is to minimize the total system costs of CCS cluster projects, including costs from CO_2_ capture, transportation, storage, and netted against revenues generated by EOR:

(Equation 1) $$\min\;f^{\pm }=\sum_{c}\left({c\underset{˜}{c}}_{c}^{\pm }+Co{\underset{˜}{m}}_{c}^{\pm }\right)\times Yc_{c}+\sum_{\left(c,j\right)}\sum_{\left(d\right)}\left(Dist_{\left(c,h\right)}^{\pm }+Dist_{\left(h,j\right)}^{\pm }\right)\times \left(t{\underset{˜}{c}}_{d}^{\pm }+To{\underset{˜}{m}}_{d}^{\pm }\right)\times Yp_{\left(c,j\right),d}+\sum_{\left(well,j\right)}\left({s\underset{˜}{c}}_{\left(well,j\right)}^{\pm }+So{\underset{˜}{m}}_{\left(well,j\right)}^{\pm }\right)\times Ys_{j}-{EOR}^{\pm }\times SE_{j}^{\pm }$$

where, $f^{\pm }$ is a full-chain cost of the CCS cluster projects under multiple uncertainties from 2030 to 2060; ${c\underset{∼}{c}}_{c}^{\pm }$ is the capital cost of CO_2_ capture for cluster c; ${Co\underset{∼}{m}}_{c}^{\pm }$ is the cumulative operation and maintenance (O&M) cost of CO_2_ capture for cluster c after discounting; ${Yc}_{c}$ is a binary variable indicating whether cluster c can be matched with storage sites via pipelines; $Dist_{\left(c,h\right)}^{\pm }$ is the total pipeline length within the cluster when the hub of cluster c is located at h; $Dist_{\left(h,j\right)}^{\pm }$ is the distance from hub h to storage site j; ${\;t\underset{∼}{c}}_{d}^{\pm }$ and ${To\underset{∼}{m}}_{d}^{\pm }$ denote the discounted capital and O&M costs per unit length of pipelines with diameter d respectively; $Yp_{\left(c,j\right),d}$ is a binary variable indicating whether cluster c can be matched with storage site j via pipelines with diameter d; ${s\underset{∼}{c}}_{\left(well,j\right)}^{\pm }$ is the capital costs of individual well modifications; ${So\underset{∼}{m}}_{\left(well,j\right)}^{\pm }$ is the cumulative storage O&M costs for storage site j after discounting; ${Ys}_{j}$ is a binary variable indicating whether storage site j is being used; ${EOR}^{\pm }$ is the average EOR revenue per ton of CO_2_ considering the predicted crude oil price, discount rate, and replacement ratio of oil to CO_2_; $SE_{j}^{\pm }$ is the cumulative CO_2_ storage amount at storage site j.

Constraints as shown in Equations 2, 3, 4, 5, 6, 7, and 8.

1. Emission reduction demands: the system is required meet the projected CCS mitigation target for the power system.

(Equation 2) $$\sum_{c}{CE}_{c}^{\pm }\times Yc_{c}\geq T^{\pm }$$

$CE_{c}^{\pm }$ is the cumulative CO_2_ capture for cluster c; $T^{\pm }$ is the projected CCS mitigation target for the power system under the carbon neutrality.

2. Storage potential: the storage potential at storage site *j* must exceed its actual cumulative CO_2_ storage quantity.

(Equation 3) $$SE_{j}^{\pm }\leq \text{cs}p_{j}^{\pm },\forall j\epsilon J$$

$csp_{j}^{\pm }$ is the storage potential at storage site *j*.

3. Cumulative capture of the cluster: the cumulative CO_2_ capture of the cluster cannot exceed the sum of the potentials of the matching storage site j.

(Equation 4) $$\sum_{c}{CE}_{c}^{\pm }\leq \sum_{j}\text{cs}p_{j}^{\pm },\forall j\epsilon N$$

4. Injection rate capacity: the annual CO_2_ storage amount at storage site j cannot exceed its injection rate capacity considering the maximum 50 injection wells.

(Equation 5) $$\frac{SE_{j}^{\pm }}{p}\leq {\text{Vin}}_{j}^{\pm }\times 50,\forall j\epsilon J$$

Where, $Vin_{j}^{\pm }$ is the single-well injection rate capacity at storage site *j*; p is the total year of the CCS technology transformation period.

5. Annual capture of the cluster: the annual capture of clusters is lower than the sum of the injection capacities of the storage sites.

(Equation 6) $$\frac{CE_{c}^{\pm }}{p}\leq \sum_{j}{\text{Vin}}_{j}^{\pm }\times 50,\forall j\epsilon N$$

6. Pipeline flow: the actual CO_2_ flow rate is lower than the upper limit of the pipeline flow with diameter *d*.

(Equation 7) $$x_{c,j}^{\pm }\leq \sum_{d}Yp_{\left(c,j\right),d}\times V_{d},\forall c\in C,j\in J,d\in D$$

Where $V_{d}$ is the upper limit of the default flow rate for a pipeline with diameter *d;* $x_{\left(c,j\right)}$ is the annual CO_2_ amount flowing from cluster c into storage site *j*.

7. Transportation distance: the CO_2_ transport distance from hub h to storage site j is limited to 250 km, as beyond which a relay station is required.

(Equation 8) $$0\leq Dist_{\left(h,j\right)}^{\pm }\leq 250$$

The nomenclature of subscripts, decision variables, and parameters is provided in Table S3. Based on the two-step method (TSM),^52^^,^^53^^,^^54^^,^^55^ this original uncertain model can be converted into two deterministic sub-models that correspond to the lower and upper bounds of the objective function value. The guiding principles are provided in Table S4. Further details can be found in Methods S3 (The CCS cluster source-sink matching model, related to STAR Methods), Figure S5, and Tables S5 and S6.

#### Data sources and processing

The geographic information in this study includes CO_2_ emission source and storage site data. The CO_2_ emission source database includes 946 power plants. It was initially derived from previous studies^26^ and was subsequently updated using the Global Power Plant Database, the Global Coal Plant Tracker,^56^ and the Carbon Emission Accounts and Datasets. Specifically, it covers operational status, spatial location, initial operating time, production capacity, energy consumption, and other technical and economic parameters. Plant-level CO_2_ emissions are calculated using the Intergovernmental Panel on Climate Change (IPCC) accounting method,^57^ ensuring consistency with internationally recognized standards. The CO_2_ storage site dataset^22^ includes information on 17 saline aquifer basins and 7 oil fields, both onshore and offshore, across China. The CO_2_ storage potential and injection capacity of all reservoirs within each basin were calculated using Darcy’s law and the US Department of Energy’s storage potential calculation method,^58^ respectively. Uncertainty due to the parameter storage efficiency is taken into account in the calculations. Considering the larger land area required for CCS clusters, the original 5 km × 5 km grid was aggregated into 40 km × 40 km using ArcGIS Desktop 10.8. These aggregated grids were used as candidate storage sites. Key economic parameters include the capital cost, O&M cost associated with CO_2_ capture at CFPPs, as well as the capital and O&M costs for pipeline transport and storage. Revenues from EOR are primarily influenced by oil prices and the CO_2_-oil displacement ratio. Detailed economic data and their sources are provided in Table S5.

Based on the CCS cluster source-sink matching model, the full chain cost of CCS technology for capture, transportation, storage, and EOR is calculated, and the cluster LCOC is further determined as shown in Equation 9.

(Equation 9) $$LCOC_{c}=\frac{{c\underset{˜}{c}}_{c}^{\pm }+{t\underset{˜}{c}}_{d}^{\pm }+{s\underset{˜}{c}}_{\left(well,j\right)}^{\pm }+\sum_{t=1}^{p}\left(Co{\underset{˜}{m}}_{c}^{\pm }+To{\underset{˜}{m}}_{d}^{\pm }+So{\underset{˜}{m}}_{\left(well,j\right)}^{\pm }\right)\times {\left(1+r\right)}^{-t}}{\sum_{t=1}^{p}{CE}_{c,t}\times {\left(1+r\right)}^{-t}},\forall c\in C,\forall j\in J,\forall d\in D$$

Further exploration is conducted on plant-level LCOC. A cost-sharing mechanism is introduced to calculate the transportation and storage costs of CFPPs. This mechanism is based on the proportion of each plant’ transport equivalents and storage amount in the corresponding part of the cluster (Equations 10, 11, 12, 13, and 14).

(Equation 10) $${LCOC}_{i}=\frac{{c\underset{˜}{i}}_{i}^{\pm }+{P\underset{˜}{i}}_{d}^{\pm }+{s\underset{˜}{i}}_{\left(well,j\right)}^{\pm }+\sum_{t=1}^{p}\left(Co{\underset{˜}{m}}_{i}^{\pm }+Po{\underset{˜}{m}}_{d}^{\pm }+So{\underset{˜}{m}}_{\left(well,j\right)}^{\pm }\right)\times {\left(1+r\right)}^{-t}}{\sum_{t=1}^{p}{CE}_{i,t}\times {\left(1+r\right)}^{-t}},\forall i\in I,\forall j\in J,\forall d\in D$$

where, ${c\underset{∼}{i}}_{i}^{\pm }{、P\underset{∼}{i}}_{d}^{\pm }\;\text{and}\;{s\underset{∼}{i}}_{\left(well,j\right)}^{\pm }$ are the capital costs of capture, transportation and storage for coal-fired power plant i, respectively; ${Co\underset{∼}{m}}_{i}^{\pm }、{Po\underset{∼}{m}}_{d}^{\pm }$ and ${So\underset{∼}{m}}_{\left(well,j\right)}^{\pm }$ denote the cumulative O&M costs for these processes; r is discount rate, which is set as 8%.

(Equation 11) $${P\underset{∼}{i}}_{d}^{\pm }={t\underset{∼}{c}}_{d}^{\pm }\times \frac{Dist_{i}^{\pm }}{Dist_{\left(c,h\right)}^{\pm }+Dist_{\left(h,j\right)}^{\pm }}$$

(Equation 12) $${Po\underset{∼}{m}}_{d}^{\pm }={To\underset{∼}{m}}_{d}^{\pm }\times \frac{Dist_{i}^{\pm }}{Dist_{\left(c,h\right)}^{\pm }+Dist_{\left(h,j\right)}^{\pm }}$$

(Equation 13) $${s\underset{∼}{i}}_{\left(well,j\right)}^{\pm }={s\underset{∼}{c}}_{\left(well,j\right)}^{\pm }\times \frac{{CE}_{i}^{\pm }}{{CE}_{c}^{\pm }}$$

(Equation 14) $${s\underset{∼}{c}}_{\left(well,j\right)}^{\pm }={So\underset{∼}{m}}_{\left(well,j\right)}^{\pm }\times \frac{{CE}_{i}^{\pm }}{{CE}_{c}^{\pm }}$$

where, $Dist_{i}^{\pm }$ is the distance from the starting point i to the next i connected to it; ${CE}_{i}^{\pm }$ is the cumulative carbon dioxide capture in plant i.

### Quantification and statistical analysis

Gurobi Optimizer 12.0.0 with Python 3.12.4 was used to perform source-sink matching simulations and basic data processing, including aggregation, classification, and sorting. Origin 2024b was used to generate statistical plots and summarize model outputs. ArcGIS Desktop 10.8.2 was used to visualize the spatial distribution of sources, sinks, and optimized transport routes.
