# Supplementary material for: Unveiling cost reduction potential of carbon capture and storage clusters in China under multiple uncertainties
Source: iScience. 2025 Aug 6;28(9):113315. doi: 10.1016/j.isci.2025.113315 (PMC12496210; doi:10.1016/j.isci.2025.113315)
Supplement: Document S1. Figures S1–S5, Tables S1–S6, and Methods S1–S3 [file mmc1.pdf]

iScience, Volume *28*

Supplemental information

Unveiling cost reduction potential of carbon capture and storage clusters in China under multiple uncertainties

Yaxian Wang, Jizhe Li, Xian Zhang, Kai Li, Jing-Li Fan, Xiaojuan Xiang, and Yunbing Hou

Supplemental Information

Contents of this file

Figure S1. Shared pipeline network of CCS clusters under multiple uncertainties, related to Figure 3.

Figure S2. Classification of clusters, related to Figure 2.

Figure S3. Framework for CCS cluster source-sink matching, related to STAR★METHODS.

Figure S4. Clustering process for coal-fired power plants (CFPPs), geographical distribution of clusters, and CO_2 capture amount, related to STAR★METHODS.

Figure S5. Shared pipeline networks of Pearl River Mouth-Guangzhou cluster, related to STAR★METHODS.

Table S1. Results of the CCS cluster source-sink matching model, related to Figure 4.

Table S2. Plant-level LCOC, related to Figure 4.

Table S3. Nomenclatures of subscripts, decision variables, and parameters, related to STAR★METHODS.

Table S4. Principle for decomposing original uncertain model into two deterministic sub-models that correspond to the lower and upper bounds of the objective function value, related to STAR★METHODS.

Table S5. Model parameters, uncertainty categorization, and data sources, related to STAR★METHODS.

Table S6. Modeling results for the Pearl River Mouth-Guangzhou cluster, related to STAR★METHODS.

Methods S1. Clustering process for coal-fired power plants and geographical distribution, related to STAR★METHODS.

Methods S2. Optimal layout for CO_2 transportation pipeline networks, related to STAR★METHODS.

Methods S3. The CCS cluster source-sink matching model, related to STAR★METHODS.

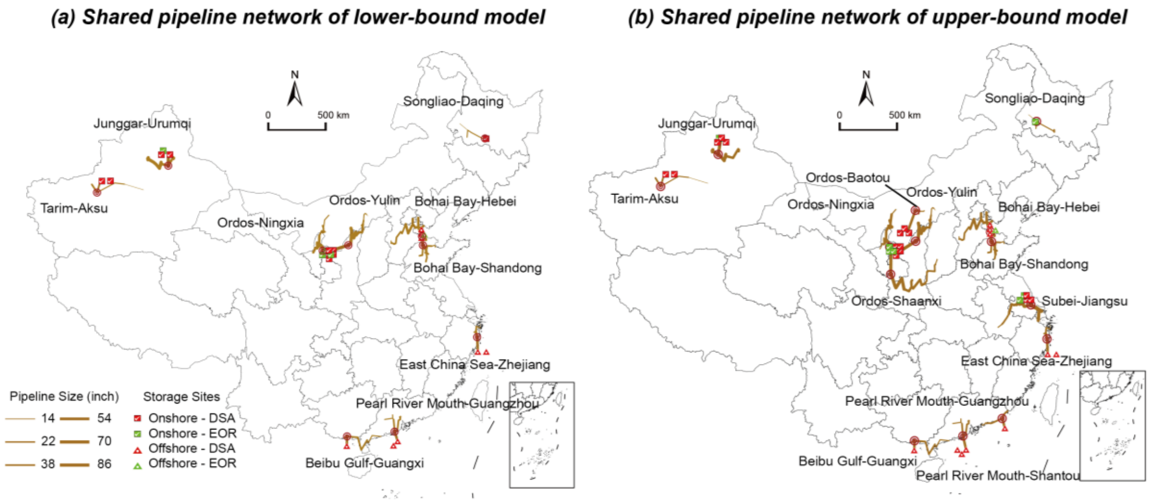

Figure S1. Shared pipeline network of CCS clusters under multiple uncertainties, related to Figure 3.

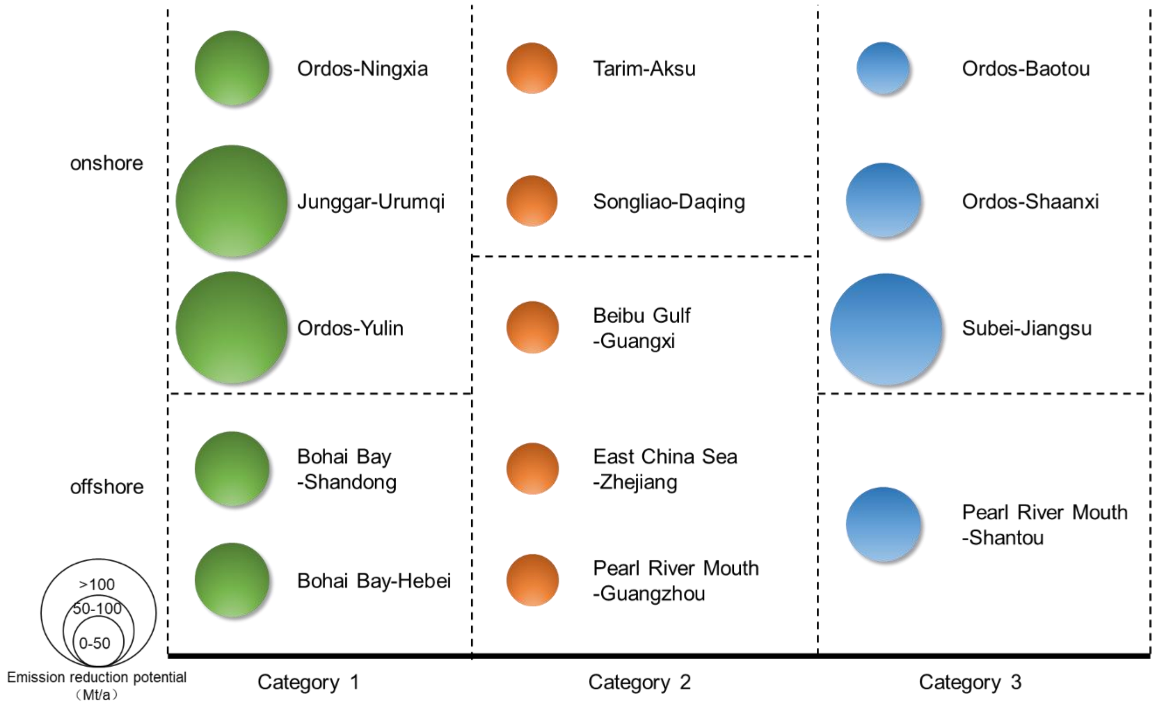

Figure S2. Classification of clusters, related to Figure 2.

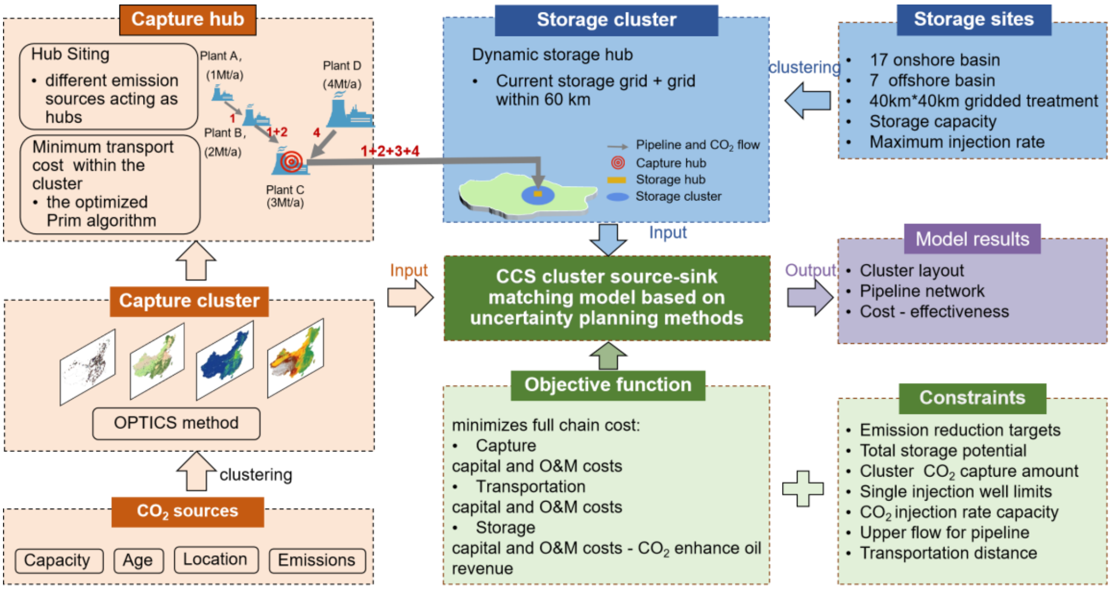

Figure S3. Framework for CCS cluster source-sink matching, related to STAR★METHODS.

Table S1. Results of the CCS cluster source-sink matching model, related to Figure 4.

Items	Results
Emission reduction target (Gt)	[19.6,32.4]
Number of clusters	[10, 14]
Number of CFPPs	[152, 222]
Cumulative emission reduction (Gt)	[19.6, 32.4]
Cumulative storage amount of DSA (Gt)	[19.5, 31.8]
Cumulative storage amount of oil field (Gt)	[0.1, 0.6]
Number of storage sites	[24, 45]
Length of cluster pipeline (km)	[5186.3, 7768.2]
Total cost (Billion USD)	[151.7, 547.9]
Capture cost (Billion USD)	[131.3, 467.4]
Transportation cost (Billion USD)	[17.6, 58.7]
Storage cost (Billion USD)	[2.8, 21.8]
Plant-level LCOC (USD/ tCO_2)	[13.6, 49.0]
LCOC of clusters (USD/ tCO_2)	[15.7, 49.2]

Table S2. Plant-level LCOC, related to Figure 4.

Cluster	Plant-level LCOC (USD/t CO_2)	Range Width (ΔLCOC)
Junggar-Urumqi	[13.8, 43.9]	30.1
Tarim-Aksu	[13.8, 30.4]	16.7
Ordos-Ningxia	[14.9, 38.5]	23.6
Ordos-Yulin	[14.8, 42.5]	27.7
Bohai Bay-Shandong	[18.3, 38.8]	20.5
Beibu Gulf-Guangxi	[16.7, 45.1]	28.3
Bohai Bay-Hebei	[17.3, 44.5]	27.2
Songliao-Daqing	[19.9, 42.0]	22.1
East China Sea-Zhejiang	[20.6, 43.8]	23.2
Pearl River Mouth-Guangzhou	[21.5, 46.0]	24.5
Ordos-Baotou	[38.0, 40.3]	2.4
Pearl River Mouth-Shantou	[44.8, 45.2]	0.4
Ordos-Shaanxi	[38.6, 46.5]	7.8
Subei-Jiangsu	[35.9, 49.0]	13.1

Table S3. Nomenclatures of subscripts, decision variables, and parameters, related to STAR★METHODS.

Symbol	Description
Subscripts	
I	Emission sources, $i \in I$
J	Storage sites, $j \in J$
C	Capture clusters, $c \in C$
D	Pipeline size (inch), $d \in D$; d = {6, 14, 22, 38, 54, 70, 86}
H	Capture hub, $h \in H$
Decision variables	
$SE_j^{\pm}$	The cumulative CO_2 storage amount at the storage site j
Yc_c	A binary variable indicating whether cluster c can be matched with storage sites via pipelines
$Yp_{(c,j),d}$	A binary variable indicating whether cluster c can be matched with storage site j via pipelines with diameter d
Ys_j	A binary variable indicating whether storage site j is being used
$f^{\pm}$	Full-chain cost of the CCS cluster projects under multiple uncertainties from 2030 to 2060
Parameters	
$T^{\pm}$	The demand for CCS cluster projects emission reductions in the power system under the carbon neutrality target (Gt)
$cc_c^{\pm}$	The capital cost of CO_2 capture (USD/kW)
$\underset{\sim}{Com}_c^{\pm}$	The cumulative O&M cost of CO_2 capture (% of capital cost)
$\underset{\sim}{tc}_d^{\pm}$	The capital cost of CO_2 transportation (USD/km) (14~86 inch)
$\underset{\sim}{Tom}_d^{\pm}$	The cumulative O&M cost of CO_2 transportation (% of capital cost)
$\underset{\sim}{sc}_{(well,j)}^{\pm}$	The capital cost of CO_2 storage (million USD/well)
$\underset{\sim}{Som}_{(well,j)}^{\pm}$	The cumulative O&M cost of CO_2 storage (% of capital cost)
$EOR^{\pm}$	Crude oil price (USD/bbl); replacement ratio of CO_2 to oil (USD)
$Dist_{(c,h)}^{\pm}$	The total length of pipelines within the cluster when the hub of cluster c is located at h
$Dist_{(h,j)}^{\pm}$	The distance from hub h to storage sites j
p	The total year of the CCS technology transformation period(year)
$CE_c^{\pm}$	The cumulative CO_2 capture for cluster c (Mt)

$csp_j^{\pm}$	The storage potential at storage site *j*
$\mathrm{Vin}_{\mathrm{j}}^{\pm}$	The single-well injection rate capacity at storage site *j*
V_d	The upper limit of the default flow rate for a pipeline with diameter d

Table S4. Principle for decomposing original uncertain model into two deterministic sub-models that correspond to the lower and upper bounds of the objective function value, related to STAR★METHODS.

Sub-models	Lower-bound model	Upper-bound model
Objective function	$Min f^{-} = \sum_{j=1}^{k} c_j^{-} x^{-} + \sum_{j=k+1}^{n} c_j^{-} x^{+}$	$Min f^{+} = \sum_{j=1}^{k} c_j^{+} x^{+} + \sum_{j=k+1}^{n} c_j^{+} x^{-}$
Constraints	$s.t. \begin{cases} \sum_{j=1}^{k} \lvert a_{ij} \rvert^{+} Sign(a_{ij}^{+}) x_j^{-} + \sum_{j=k+1}^{n} \lvert a_{ij} \rvert^{-} Sign(a_{ij}^{-}) x_j^{+} \leq b_i^{-}, \quad i = 1,2 \ldots m \\ x^{-} \geq 0 \end{cases}$	$s.t. \begin{cases} \sum_{j=1}^{k} \lvert a_{ij} \rvert^{-} Sign(a_{ij}^{-}) x_j^{+} + \sum_{j=k+1}^{n} \lvert a_{ij} \rvert^{+} Sign(a_{ij}^{+}) x_j^{-} \leq b_i^{+}, \quad i = 1,2 \ldots m \\ x^{+} \geq 0 \end{cases}$
Decomposition principle	**Most optimistic strategy:**<br>Objective function: all parameters are set to be the minimum values;<br>Constraints: loose	**Most conservative strategy:**<br>Objective function: all parameters are set to be the maximum values;<br>Constraints: tight

Table S5. Model parameters, uncertainty categorization, and data sources, related to STAR★METHODS.

Categorization of uncertainty	Items				Values	Data sources
Policy	The demand for CCS cluster projects emission reductions in the power system under the carbon neutrality target (Gt)				[19.6, 32.4]	[1,2]
Technical	Storage potential (Gt)	DSA			[1894.2, 3198.6]	[3]
		EOR			[8.0, 8.6]	
Economic	CO_2 capture	Capital cost (USD/kW)			[464, 1000]	[4,5]
		Annual O&M cost (% of capital cost)			[3.7, 4.0]	[5,6]
	CO_2 transportation	Capital cost (million USD/km)	Onshore pipeline (inch)	6	[0.1, 0.1]	[7-9]
				14	[0.3, 0.6]	
				22	[0.8, 1.5]	
				38	[2.3, 4.4]	
				54	[4.5, 8.6]	
				70	[7.3, 14.0]	
				86	[10.9, 20.8]	
			Offshore pipeline (inch)	6	[0.1, 0.2]	
				14	[0.5, 0.9]	
				22	[1.2, 2.0]	
				38	[3.8, 6.1]	
				54	[7.3, 11.8]	
				70	[11.9, 22.6]	
				86	[17.6, 19.3]	
		Annual O&M cost (% of capital cost)	Onshore		[2.5, 10.0]	[10]
			Offshore		[3.0, 8.0]	[11]

	CO_2 storage	Capital cost (million USD/well)	Onshore	[135, 500]	12-14
			Offshore	[435, 1500]	
		Annual O&M cost (% of capital cost)		30	14
	CO_2-EOR	Crude oil price (USD/bbl); replacement ratio of CO_2 to oil (USD)		[35, 70]; 4:1	15,16

Methods S1. Clustering process for coal-fired power plants and geographical distribution, related to STAR★METHODS.

For CCS, the concept of clusters takes advantage of the fact that many CO_2 emitting facilities are geographically concentrated[17]. This study aims to identify potential CCS clusters in China by first screening the emission sources and then grouping geographically neighboring sources into clusters using an appropriate clustering algorithm.

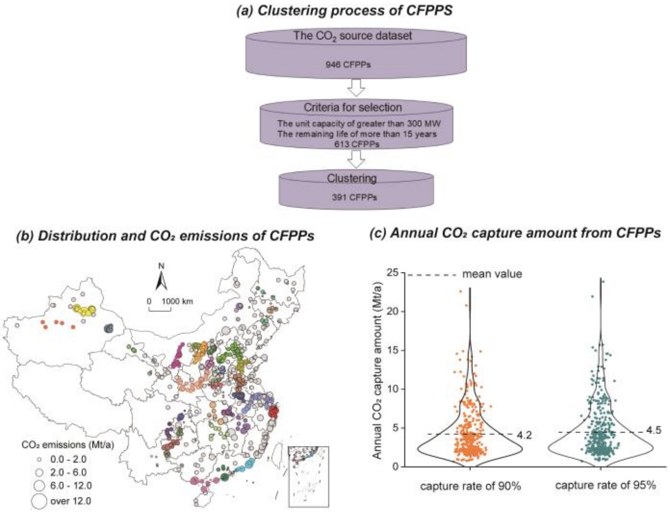

Figure S4. Clustering process for coal-fired power plants (CFPPs), geographical distribution of clusters, and CO_2 capture amount, related to STAR★METHODS. *Note:* Colored dots in figure (b) denote different clusters, and grey dots indicate data points that do not belong to any cluster.

As shown in Figure S4. (a), the CO_2 source dataset includes a total of 946 CFPPs (no data for Hong Kong, Macao, and Taiwan). To ensure the economic feasibility of CCS retrofitting, plants with an installed capacity of less than 300 MW and a remaining operating life of less than 15 years were excluded. This screening identified 613 CFPPs as suitable candidates for clustering.

Although K-means is widely used[18], it exhibits notable limitations in delineating CCS clusters. It requires a predefined number of clusters and tends to form spherical clusters, making it ill-suited to the irregular spatial distribution of emission sources. More critically, it lacks the ability to exclude geographically dispersed outliers, which may compromise cluster economics and hinder the optimization of transport infrastructure. The OPTICS (Ordering Points to Identify the Clustering Structure) algorithm[19] does not require a predefined number of clusters and can identify clusters of arbitrary shapes. It also filters out discrete points, thereby improving the accuracy and practicality of cluster delineation. The algorithm is based on the concept of density reachability and relies on three key parameters. These are: the minimum number of samples in a neighborhood (minSamples) to define core points, the minimum cluster size (minClusterSize) to constrain intra-cluster sample counts, and the steepness parameter (xi) to detect significant changes in the reachability plot for extracting multi-density cluster structures.

Accordingly, this study employed the sklearn.cluster.OPTICS module in the Python 3.12.4 to perform spatial clustering on 613 candidate emission sources. Based on preliminary results[20] and considering the total number of sources and silhouette scores, the parameters were set to *minSamples* = 5, *minClusterSize* = 0.01, and *xi* = 0.06. This configuration yielded 36 clusters, with an average annual CFPP capture volume of [4.2, 4.5] Mt/a (Figure S4. (b) and (c)). The clusters with a dense distribution in Northwest, North, East, and Southwest China regions. Large-scale CO_2 emitters are primarily concentrated in Northwest, North, and South China regions.

Methods S2. Optimal layout for CO_2 transportation pipeline networks, related to STAR★METHODS.

The optimal layout of the CO_2 transport network can be determined using graph theory algorithms. In graph theory, a tree T is defined as a connected graph without cycles $G = (V, E)$, where V is the number of vertices (nodes), E is the number of edges (links), and there is exactly one path between any two nodes.

To minimize the full-chain cost of CCS clusters and ensure transparent deployment of pipeline networks, a hub-sensitive minimum cost spanning tree (MST) algorithm with hub selection is proposed. This approach builds on cluster characteristics and prior studies. A distinctive feature of this algorithm is that pipeline transportation costs are explicitly incorporated as edge weights.

The core logic is as follows:

(1) Assume that the hub can be sited at any emission source within the cluster;

(2) Calculate the pipeline transportation cost for each edge and construct the graph $G = (V, E, W)$. Set the vertex set V (emission sources in the cluster); calculate the distance between all nodes (emission sources) in the cluster and set it to the set E; increase the pipeline transportation cost as the edge weight w, which is related to the pipeline size and transportation distance. The pipeline size needs to be chosen based on the dynamic transportation volume, which is the sum of the transportation volume of that segment of the pipeline and all upstream pipelines. From this, a graph G is constructed;

(3) For each hub location, we use the Prim algorithm to construct a minimum spanning tree (MST);

(4) Traverse all possible hub locations within the cluster, compute the total transportation cost for each case, and record the results.;

(5) Traverse the MST in reverse in order to derive the connection paths between nodes, pipe diameters and associated costs.

This approach accounts not only for spatial distance and network connectivity but also integrates hub location optimization and a segmented cost mechanism, thereby simulating the real-world construction of a hierarchical pipeline network. In contrast to conventional models that place hubs at geometric centroids, this method offers a more accurate representation of cost heterogeneity in infrastructure planning.

Methods S3. The CCS cluster source-sink matching model, related to STAR★METHODS.

The CCS cluster source-sink matching model can be simplified to an interval constrained mathematical programming problem. Interval Linear Programming (ILP) can handle uncertain information in the form of intervals, generally of the form:

$$Min f^{\pm} = C^{\pm} X^{\pm} \quad (1a)$$

subject to:

$$A^{\pm} X^{\pm} \leq B^{\pm} \quad (1b)$$

$$X^{\pm} \geq 0 \quad (1c)$$

Where $A^{\pm} \in \{R^{\pm}\}^{m \times n}$, $C^{\pm} \in \{R^{\pm}\}^{1 \times n}$, $B^{\pm} \in \{R^{\pm}\}^{m \times 1}$, $X^{\pm} \in \{R^{\pm}\}^{n \times 1}$; $R^{\pm}$ denotes a set of interval numbers; $A^{\pm} = (a_{ij}^{\pm})_{m \times n}$, $C^{\pm} = (c_1^{\pm}, c_2^{\pm}, \ldots, c_n^{\pm})$, $B^{\pm} = (b_1^{\pm}, b_2^{\pm}, \ldots, b_m^{\pm})^T$ and $X^{\pm} = (x_1^{\pm}, x_2^{\pm}, \ldots, x_n^{\pm})^T$. An interval number $a^{\pm}$ is defined as $a^{\pm} = [a^-, a^+] = \{t \in a | a^- \leq t \leq a^+\}$.

When the system's goal and constraints are fuzzy, model (1) can be converted into an interval-fuzzy problem, through incorporating fuzzy-programming concepts within the ILP framework[23,24]. Thus, an interval-parameter fuzzy linear programming model can be formulated as follows:

$$\underset{\sim}{min} f^{\pm} = \sum_{j=1}^{k} \underset{\sim}{c}_j^{\pm} x_j^{\pm} + \sum_{j=k+1}^{n} \underset{\sim}{c}_j^{\pm} x_j^{\pm} \quad (2a)$$

subject to:

$$\sum_{j=1}^{k} a_{ij}{}^{\pm} x_j^{\pm} + \sum_{j=k+1}^{n} a_{ij}{}^{\pm} x_j^{\pm} \leq b_j^{\pm}, i = 1,2 \ldots m \quad (2b)$$

$$0 \le x_j^{\pm}, j = 1,2,..n \tag{2c}$$

Where $a_{ij}{}^{\pm}$, $b_j{}^{\pm}$, $\underset{\sim}{c}_j^{\pm}$ and $x_j^{\pm}$ represent interval parameters, $x_j^{\pm}$ denotes the decision variables, and $\underset{\sim}{c}_j^{\pm}$ corresponds to interval parameters with fuzzy boundaries. Assuming $b_j^{\pm} > 0, \mathrm{f}^{\pm} > 0$, $\underset{\sim}{c}_j^{\pm}$ and $a_{ij}{}^{\pm}$ are non-negative coefficients for $j = 1,2, \ldots k$; while $\underset{\sim}{c}_j^{\pm}$ and $a_{ij}{}^{\pm}$ become negative coefficients for $j = k + 1, \mathrm{k} + 2, \ldots n$. The triangular fuzzy membership function is characterized by its center c^c and the distance γ, from any given point to center, denoted as $\underset{\sim}{C} = (c^c, \gamma)$. Under linear conditions, this membership function, $\mu_c(x)$ can be expressed as:

$$\mu_c(x) = \begin{cases} 0, \ if x < c^c - \gamma \ or \ x > c^c + \gamma \\ 1 - \frac{|c^c - x|}{\gamma}, \ if c^c - \gamma \le x \le c^c + \gamma \end{cases} \tag{2d}$$

Equation (2a) can be transformed into:

$$\underset{\sim}{min f}^{\pm} = (\sum_{j=1}^{\mathrm{k}} \underset{\sim}{c}_j^{\pm} x_j^{\pm} + \sum_{j=k+1}^{n} \underset{\sim}{c}_j^{\pm} x_j^{\pm}, \sum_{j=1}^{\mathrm{k}} \gamma_j |x_j^{\pm}| + \sum_{j=k+1}^{n} \gamma_j |x_j^{\pm}|) \tag{2e}$$

subject to:

$$\sum_{j=1}^{\mathrm{k}} a_{ij}{}^{\pm} x_j^{\pm} + \sum_{j=k+1}^{n} a_{ij}{}^{\pm} x_j^{\pm} \le \mathrm{b}_{\mathrm{j}}^{\pm}, i = 1,2 \ldots m \tag{2f}$$

$$0 \le x_j^{\pm}, j = 1,2,..n \tag{2g}$$

An interactive two-step solution algorithm for solving model (2) is provided by Huang et al[25], The lower-bound sub-model f^- can be expressed as:

$$min f^- = \sum_{j=1}^{\mathrm{k}} c_j^- x_j^- + \sum_{j=1}^{k} \gamma_j |x_j^-| + \sum_{j=k+1}^{n} c_j^- x_j^+ + \sum_{j=k+1}^{n} \gamma_j |x_j^+| \tag{3a}$$

subject to:

$$\sum_{j=1}^{\mathrm{k}} |a_{ij}|^+ Sign(a_{ij}^+) x_j^- + \sum_{j=k+1}^{n} |a_{ij}|^- Sign(a_{ij}^-) x_j^+ \le b_i^-, i = 1,2 \ldots m \tag{3b}$$

$$0 \le x_j^-, j = 1,2,..k \tag{3c}$$

$$0 \le x_j^+, j =, k + 1, k + 2,..n \tag{3d}$$

Solutions of $f_{jopt}^-, x_{jopt}^-, (j = 1,2 \ldots k)$ and $x_{jopt}^+, (j = k + 1, k + 2, \ldots, n)$ can be obtained through solving sub-model (2). Based on the solutions of sub-model (2), the sub-model corresponding to f^+ can be formulated as follows (assuming that $b_i^{\pm} > 0$ and $f^{\pm} > 0$):

$$min f^+ = \sum_{j=1}^{\mathrm{k}} c_j^+ x_j^+ + \sum_{j=1}^{\mathrm{k}} \gamma_j |x_j^+| + \sum_{j=k+1}^{n} c_j^+ x_j^- + \sum_{j=k+1}^{n} \gamma_j |x_j^-| \tag{4a}$$

$$\sum_{j=1}^{\mathrm{k}} |a_{ij}|^- Sign(a_{ij}^-) x_j^+ + \sum_{j=k+1}^{n} |a_{ij}|^+ Sign(a_{ij}^+) x_j^- \le b_i{}^+, i = 1,2 \ldots m \tag{4b}$$

$$x_j^+ \ge x_{jopt}^-, j = 1,2, \ldots k \tag{4c}$$

$$x_{jopt}^+ \ge x_j^- \ge 0, j = k + 1, k + 2, \ldots n \tag{4d}$$

Solutions of $f_{jopt}^+, x_{jopt}^+, (j = 1,2 \ldots k)$ and $x_{jopt}^-, (j = k + 1, k + 2, \ldots, n)$ can be obtained through solving sub-model (3). The model-solving procedure is as follows:

(1) The objective function and constraints are transformed into an interval programming problem using the interval fuzzy programming approach;

(2) The interval linear programming problem is solved by decomposing the model into a lower-bound sub-model, yielding the solution f_{opt}^-;

(3) The solution of the lower-bound sub-model is incorporated as a constraint to construct the upper-bound sub-model, which is then solved to obtain f_{opt}^+;

(4) The results of the upper and lower bound sub-models are integrated to derive the optimal solution of the model: the total cost is expressed as $f_{opt}^{\pm} = [f_{opt}^{-}, f_{opt}^{+}]$, and the decision variables are expressed as $x_{jopt}^{\pm} = [x_{jopt}^{-}, x_{jopt}^{+}]$.

In order to solve the CCS cluster source-sink matching model based on fuzzy possibility programming and interval linear programming methods, the original interval linear programming model can be converted into two sub-models which correspond to the lower and upper bounds of the objective-function value. The interactive two-step solution algorithm developed by Huang et al.[24] is applied in this model. The two sub-models are presented as follows. Firstly, the lower-bound sub-model is constructed:

$$minf^{-} = \sum_c (\underset{\sim}{cc_c^{-}} + \underset{\sim}{Com_c^{-}}) \times Yc_c + \sum_{(c,j)} \sum_d \left(Dist_{(c,h)}^{-} + Dist_{(h,j)}^{-}\right) \times (\underset{\sim}{tc_d^{-}} + \underset{\sim}{Tom_d^{-}}) \times Yp_{(c,j),d} + \sum_{(well,j)} (\underset{\sim}{sc_{(\text{well,j})}^{-}} + \underset{\sim}{Som_{(well,j)}^{-}}) \times Ys_j - EOR^{-} \times SE_j^{+} \tag{5a}$$

$$s.t.\begin{cases} \sum_c CE_c^{-} \times Yc_c \geq T^{-} \\ SE_j^{-} \leq \text{csp}_\text{j}^{+}, \forall j \epsilon J \\ \sum_c CE_c^{-} \leq \sum_j \text{csp}_\text{j}^{+}, \forall j \epsilon N \\ \frac{SE_j^{-}}{p} \leq \text{Vin}_\text{j}^{+} \times 50, \forall j \epsilon J \\ \frac{CE_c^{-}}{p} \leq \sum_j Vin_j^{+} \times 50, \forall j \epsilon N \\ x_{c,j}^{+} \leq \sum_d Yp_{(c,j),d} \times \text{V}_d, \forall c \in C, j \in J, d \in D \\ 0 \leq Dist_{(c,j)}^{-} \leq 250 \end{cases} \tag{5b}$$

Solutions of $(SE_j^{+})_{opt}$, $(x_{c,j}^{+})_{opt}$, $(Yc_c)_{opt}$, $(Yp_{(c,j),d})_{opt}$, $(Ys_j)_{opt}$, $(Yo_j)_{opt}$, and f_{opt}^{-} can be obtained through solving sub-model (5). Next, construct the upper-bound sub-model (6) based on these optimized decision variables, and get the solutions of the sub-model f_{opt}^{+}.

$$minf^{+} = \sum_c (\underset{\sim}{cc_c^{+}} + \underset{\sim}{Com_c^{+}}) \times Yc_c + \sum_{(c,j)} \sum_d \left(Dist_{(c,h)}^{+} + Dist_{(h,j)}^{+}\right) \times \left(\underset{\sim}{tc_d^{+}} + \underset{\sim}{Tom_d^{+}}\right) \times Yp_{(c,j),d} + \sum_{(well,j)} (\underset{\sim}{sc_{(\text{well,j})}^{+}} + \underset{\sim}{Som_{(well,j)}^{+}}) \times Ys_j - EOR_j^{+} \times SE_j^{-} \tag{6a}$$

$$s.t.\begin{cases} \sum_c CE_c^{+} \times Yc_c \geq T^{+} \\ SE_j^{+} \leq \text{csp}_\text{j}^{-}, \forall j \epsilon J \\ \sum_c SE_c^{+} \leq \sum_j csp_j^{-}, \forall j \epsilon N \\ \frac{SE_j^{+}}{p} \leq \text{Vin}_\text{j}^{-} \times 50, \forall j \epsilon J \\ \frac{CE_c^{+}}{p} \leq \sum_j Vin_j^{-} \times 50, \forall j \epsilon N \\ x_{c,j}^{-} \leq \sum_d Yp_{(c,j),d} \times \text{V}_d, \forall c \in C, j \in J, d \in D \\ 0 \leq Dist_{(c,j)}^{+} \leq 250 \\ \text{SE}_\text{j}^{-} \leq (SE_j^{+})_{opt} \end{cases} \tag{6b}$$

Solutions of $(SE_j^{-})_{opt}$, $(x_{c,j}^{-})_{opt}$, $(Yc_c)_{opt}$, $(Yp_{(c,j),d})_{opt}$, $(Ys_j)_{opt}$, $(Yo_j)_{opt}$, and f_{opt}^{+} can be obtained through solving sub-model (6). Thus, the final solutions of the CCS cluster source-sink matching model can be obtained as $x_{c,j}^{\pm} = [x_{c,j}^{-}, x_{c,j}^{+}]$, $SE_j^{\pm} = [SE_j^{-}, SE_j^{+}]$, and $f_{opt} = [f_{opt}^{-}, f_{opt}^{+}]$.

Guangdong Province is the first in China to release a provincial CCS development roadmap and has accumulated extensive experience in offshore storage assessment and regulatory development. To illustrate the impact of uncertainty on CCS cluster layout and cost assessment, we selected the Pearl River Mouth-Guangzhou cluster, which includes 17 coal-fired power plants, as a representative case study. Figure S5 compares the shared pipeline networks of this cluster under the lower- and upper-bound models. The hub locations remain consistent across both scenarios, indicating a degree of robustness in site selection. However, the upper-bound model shows a significant increase in CO_2 capture amount, required storage sites, and pipeline length (Table S6). Notably, the total system cost varies within the range of [11.87, 26.35]

billion USD, highlighting the significant influence of uncertainties on CCS deployment costs.

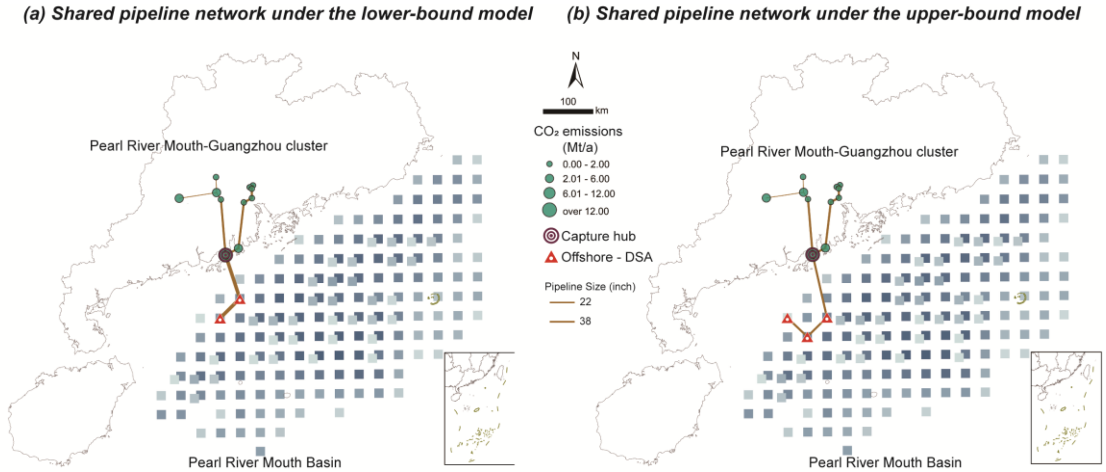

Figure S5. Shared pipeline networks of Pearl River Mouth-Guangzhou cluster, related to STAR★METHODS.

Table S6. Modeling results for the Pearl River Mouth-Guangzhou cluster, related to STAR ★METHODS.

Items	Model results
Annual CO_2 capture (Mt/a)	[42.4, 44.7]
Cumulative CO_2 capture (Mt)	[1313.6, 1386.6]
Storage sites	[2, 3]
Pipeline length (km)	[539.8, 606.9]
Total cost (billion USD)	[11.87, 26.35]

References

1. ACCA 21 (2024). Annual report on carbon dioxide capture, utilization, and storage (CCUS) in China (2024). Internal Report.
2. Zhang, X., Yang, X., Lu, X., Chen, J., Cheng, J., Diao, Y., Fan, J., Gao, L., Gao, S., and Han, L. (2023). Annual report on carbon dioxide capture, utilization, and storage (CCUS) in China (2023). https://www.acca21.org.cn/trs/000100170002/16690.html.
3. Fan, J.-L., Xiang, X., Yao, Y., Li, K., Li, Z., Wei, S., Diao, Y., Ju, Z., Li, X., Li, X., et al. (2025). Dataset of CO_2 geological storage potential and injection rate capacity in China based on fine grid technology. Scientific Data *12*, 640. https://doi.org/10.1038/s41597-025-04875-3.
4. IEAGHG (2019). Towards zero-emission carbon capture and storage in power plants using higher capture rates or biomass. https://climit.no/app/uploads/sites/4/2019/09/IEAGHG-Report-2019-02-Towards-zero-emissions.pdf.
5. Chen, W., Lu, X., Lei, Y., and Chen, J.-F. (2021). A Comparison of Incentive Policies for the Optimal Layout of CCUS Clusters in China's Coal-Fired Power Plants Toward Carbon Neutrality. Engineering *7*, 1692-1695. https://doi.org/10.1016/j.eng.2021.11.011.
6. Yang, L., Xu, M., Yang, Y., Fan, J., and Zhang, X. (2019). Comparison of subsidy schemes for carbon capture utilization and storage (CCUS) investment based on real option approach: Evidence from China. Applied Energy *255*, 113828. https://doi.org/10.1016/j.apenergy.2019.113828.
7. Tian, Q., Zhao, D., Li, Z., and Zhu, Q. (2017). Robust and stepwise optimization design for CO_2 pipeline transportation. International Journal of Greenhouse Gas Control *58*, 10-18. https://doi.org/10.1016/j.ijggc.2017.01.003.
8. Sanchez, D.L., Johnson, N., McCoy, S.T., Turner, P.A., and Mach, K.J. (2018). Near-term deployment of carbon capture and sequestration from biorefineries in the United States. Proceedings of the National Academy of Sciences *115*, 4875-4880. https://doi.org/1

0.1073/pnas.1719695115.
9. Dou, L., Sun, L., Lyu, W., Wang, M., Gao, F., Gao, M., and Jiang, H. (2023). Trend of global carbon dioxide capture, utilization and storage industry and challenges and countermeasures in China. Petroleum Exploration and Development *50*, 1246-1260. https://doi.org/10.1016/S1876-3804(23)60463-X.
10. Chen, C., Ma, S., Wang, X., Shen, J., Qin, Y., Ling, Z., and Song, Y. (2024). CCUS source-sink matching model based on sink well placement optimization. Fuel *377*, 132812. https://doi.org/10.1016/j.fuel.2024.132812.
11. Calvillo, C., Race, J., Chang, E., Turner, K., and Katris, A. (2022). Characterisation of UK Industrial Clusters and Techno-Economic Cost Assessment for Carbon Dioxide Transport and Storage Implementation. International Journal of Greenhouse Gas Control *119*, 103695. https://doi.org/10.1016/j.ijggc.2022.103695.
12. Wei, N., Li, X., Dahowski, R.T., Davidson, C.L., Liu, S., and Zha, Y. (2015). Economic evaluation on CO_2-EOR of onshore oil fields in China. International Journal of Greenhouse Gas Control *37*, 170-181. https://doi.org/10.1016/j.ijggc.2015.01.014.
13. LIU Muxin, L.X., LIN Qianguo (2021). Economic analysis and risk assessment for carbon capture, utilization and storage project under the background of carbon neutrality in China. Thermal Power Generation *50*, 18-26. https://doi.org/10.19666/j.rlfd.202101009.
14. GCCSI (2021). Technology Readiness and Costs of CCS. https://www.globalccsinstitute.com/resources/publications-reports-research/technology-readiness-and-costs-ofccs/.
15. Sun, L., Liu, Q., Chen, H., Yu, H., Li, L., Li, L., Li, Y., and Adenutsi, C.D. (2024). Source-sink matching and cost analysis of offshore carbon capture, utilization, and storage in China. Energy *291*, 130137. https://doi.org/10.1016/j.energy.2023.130137.
16. Fan, J.-L., Li, Z., Ding, Z., Li, K., and Zhang, X. (2023). Investment decisions on carbon capture utilization and storage retrofit of Chinese coal-fired power plants based on real option and source-sink matching models. Energy Economics *126*, 106972. https://doi.org/10.1016/j.eneco.2023.106972.
17. GCCSI (2016). The Global Status of CCS. Special Report: Understanding Industrial CCS Hubs and Clusters. https://www.globalccsinstitute.com/wp-content/uploads/2019/08/Understanding-Industrial-CCS-hubs-and-clusters.pdf.
18. Rodriguez, A., and Laio, A. (2014). Clustering by fast search and find of density peaks. Science *344*, 1492-1496. https://doi.org/10.1126/science.1242072.
19. Liu, R., Wang, H., and Yu, X. (2018). Shared-nearest-neighbor-based clustering by fast search and find of density peaks. Information Sciences *450*, 200-226. https://doi.org/10.1016/j.ins.2018.03.031.
20. Li, J. (2024). Research on the planning of multi-industry CCUS cluster shared pipeline network in China under the background of carbon neutrality. Maste thesis (China University of Mining and Technology (Beijing)).
21. GCCSI (2024). Global Status of CCS 2024 Report. www.globalccsinstitute.com/global-status-report/.
22. CCSA (2013). CCS Cost Reduction Task Force: Final Report. https://assets.publishing.service.gov.uk/government/uploads/system/uploads/attachment_data/file/201021/CCS_Cost_Reduction_Taskforce_-_Final_Report_-_May_2013.pdf.
23. Huang, Y.F., Baetz, B.W., Huang, G.H., and Liu, L. (2002). Violation analysis for solid waste management systems: an interval fuzzy programming approach. Journal of Environmental Management *65*, 431-446. https://doi.org/10.1006/jema.2002.0566.
24. Huang, G.H., Baetz, B.W., and Patry, G.G. (1992). A gray linear-programming approach for municipal solid-waste management planning under uncertainty. Civil Engineering Systems *9*, 319-335. https://doi.org/10.1080/02630259208970657.
25. Huang, G.H., Baetz, B.W., and Patry, G.G. (1993). A grey fuzzy linear programming approach for municipal solid waste management planning under uncertainty. Civil Engineering Systems *10*, 123-146. https://doi.org/10.1080/02630259308970119.
